# Augmented Reality in Ophthalmology

**DOI:** 10.22336/rjo.2025.73

**Published:** 2025

**Authors:** Consuela-Mădălina Gheorghe

**Affiliations:** PhD, Philologist, Certified translator

Rapid advancements in augmented reality (AR) have led to its application in a variety of industries, including cultural heritage, maintenance, and medicine. Since most AR systems are based on visual systems, ophthalmology has a closer relationship with AR than other fields (Li T, Li C, Zhang X, Liang W, Chen Y, Ye Y, Lin H. Augmented Reality in Ophthalmology: Applications and Challenges. Front Med (Lausanne). 2021 Dec 10;8:733241. doi: 10.3389/fmed.2021.733241. PMID: 34957138; PMCID: PMC8703032).

The technology known as augmented reality (AR) adds real-time computer-generated information to the natural world (Carmigniani J, Furht B, Anisetti M, Ceravolo P, Damiani E, Ivkovic M. Augmented reality technologies, systems and applications. Multimed Tools Appl. (2011) 51:341–77. 10.1007/s11042-010-0660-6).

AR is not limited to visual perception. It can be used to all senses, including touch, hearing, smell, and sight (Azuma R, Baillot Y, Behringer R, Feiner S, Julier S, MacIntyre B. Recent advances in augmented reality. IEEE Comput Graph Appl. (2001) 21:34–47. 10.1109/38.96345927295638). AR applications include eliminating or processing real-world objects, as well as adding virtual information to them; this is known as mediated reality or reduced reality (Carmigniani J, Furht B, Anisetti M, Ceravolo P, Damiani E, Ivkovic M. Augmented reality technologies, systems and applications. Multimed Tools Appl. (2011) 51:341–77. 10.1007/s11042-010-0660-6; Azuma R, Baillot Y, Behringer R, Feiner S, Julier S, MacIntyre B. Recent advances in augmented reality. IEEE Comput Graph Appl. (2001) 21:34–47. 10.1109/38.96345927295638). Unlike virtual reality (VR), which completely immerses users in a computer-generated virtual world, AR is based on the natural world and enhances the real environment with computer-generated information (Carmigniani J, Furht B, Anisetti M, Ceravolo P, Damiani E, Ivkovic M. Augmented reality technologies, systems and applications. Multimed Tools Appl. (2011) 51:341–77. 10.1007/s11042-010-0660-6).

Thus, it can be stated that Ophthalmology is a field at the core of digital innovation and patient-centered care, with a foundation in precise imaging, microsurgery, and real-time decision-making. Incorporating augmented reality (AR) technologies into ophthalmic practice represents not only a technological advancement but also a potential paradigm shift in how eye care is provided, taught, and experienced.

By superimposing digital data onto the real visual field, augmented reality enhances perception without replacing reality. The clinical operations in ophthalmology, which now rely on layered data—imaging, measurements, and surgical plans—processed in real time, are well suited to this functionality. While virtual reality completely immerses the user in a simulated environment, augmented reality preserves situational awareness, which is crucial for both clinical evaluation and operation.

One of the most promising applications of augmented reality in ophthalmology is surgical guidance. In vitreoretinal and anterior segment surgery, where millimeter-scale accuracy influences outcomes, AR-enabled heads-up display devices allow surgeons to view the operative field with imaging data, such as OCT, angiography, or preoperative planning maps. By projecting this data directly into the surgeon‘s field of view, AR reduces the need for them to switch between the microscope and external monitors, potentially improving focus, ergonomics, and procedural efficiency (Broderick RC, Spurzem GJ, Jeffery Reeves J, Hollandsworth HM, Sandler BJ, Jacobsen GR, Longhurst CA, Horgan S. First use of augmented reality headset in minimally invasive general surgery: seeing is believing. Surg Endosc. 2025 Sep;39(9):6055-6060. doi: 10.1007/s00464-025-11985-x. Epub 2025 Jul 10. PMID: 40640628; PMCID: PMC12408766). Preliminary studies suggest that these tools could shorten learning curves and improve surgical accuracy, particularly in difficult cases.

Another area that has a significant impact is ophthalmic education and training. Apprenticeship programs and restricted access to high-risk procedures are key components of traditional surgical training (Riddle EW, Kewalramani D, Narayan M, Jones DB. Surgical Simulation: Virtual Reality to Artificial Intelligence. Current Problems in Surgery. 2024;61(11):101625. doi: https://doi.org/10.1016/j.cpsurg.2024.101625). Thanks to AR-based simulators and coaching tools, trainees can practice procedures with real-time visual cues, anatomical overlays, and performance feedback. These technologies make a more reliable, reproducible, and safe learning environment possible. By allowing seasoned surgeons to mentor less-experienced colleagues across geographic boundaries, AR-enabled remote mentoring may help fill gaps in specialized training in resource-limited settings (Anyinkeng AB, Girma SM, Maurice T, JohnPaul E, Hiwot T, Awad AK). The role of remote and virtual surgical training in expanding cardiothoracic surgical capacity in low-resource regions. BMC Surg. 2025 Aug 25;25(1):393. doi: 10.1186/s12893-025-03142-x. PMID: 40855426; PMCID: PMC12376457).

AR is becoming increasingly crucial for diagnostics and clinical decision support outside the operating room (Lastrucci A, Wandael Y, Barra A, Ricci R, Maccioni G, Pirrera A, Giansanti D. Exploring Augmented Reality Integration in Diagnostic Imaging: Myth or Reality?). Diagnostics (Basel). 2024 Jun 23;14(13):1333. doi: 10.3390/diagnostics14131333. PMID: 39001224; PMCID: PMC11240696). Overlaying visual field data, corneal mapping, or retinal imaging onto in-person patient assessments would increase diagnostic accuracy and streamline workflow (Montesano G, Lazaridis G, Ometto G, Crabb DP, Garway-Heath DF). Improving the Accuracy and Speed of Visual Field Testing in Glaucoma With Structural Information and Deep Learning. Transl Vis Sci Technol. 2023 Oct 3;12(10):10. doi: 10.1167/tvst.12.10.10. PMID: 37831447; PMCID: PMC10587851). For example, in glaucoma treatment, AR systems could instantly integrate structural and functional data into the clinician‘s field of vision, allowing for more complex assessments of the disease‘s progression (Tonti E, Tonti S, Mancini F, Bonini C, Spadea L, D‘Esposito F, Gagliano C, Musa M, Zeppieri M. Artificial Intelligence and Advanced Technology in Glaucoma: A Review. J Pers Med. 2024 Oct 16;14(10):1062. doi: 10.3390/jpm14101062. PMID: 39452568; PMCID: PMC11508556). Similarly, in planning refractive surgery, AR may improve alignment between preoperative measurements and intraoperative execution (Shen Y, Wang S, Shen Y, Hu J. The Application of Augmented Reality Technology in Perioperative Visual Guidance: Technological Advances and Innovation Challenges. Sensors. 2024; 24(22):7363. https://doi.org/10.3390/s24227363).

Additionally, AR has the potential to improve patient communication and engagement—two aspects of ophthalmic treatment that are frequently disregarded (Khandekar A, Shah A, Miranda LAE, Bello FT, Liebl B, Ryan J, de Paula PAB, Bhatia A, Guerard T, Parekh DJ. Augmented Reality in Outpatient Care: A Narrative Review. Healthc Technol Lett. 2025 Nov 22;12(1):e70025. doi: 10.1049/htl2.70025. PMID: 41287787; PMCID: PMC12640284). Patients can have a better understanding of their condition and take an active role in decision-making by using augmented reality (AR) to see disease processes, treatment strategies, or surgical outcomes. AR-based visual simulations may enhance informed consent and align patient expectations with clinical realities for disorders such as cataracts, macular degeneration, or refractive problems.

Even with these advantages, there are still difficulties in using AR in ophthalmology. Technical constraints, such as latency, resolution restrictions, and system interoperability with existing equipment, remain significant barriers. In surgical settings, even little delays or incorrect picture alignment could compromise patient safety. Therefore, rigorous validation, standardization, and regulatory oversight are essential before widespread application.

Accessibility and cost are two further issues. Hardware, software, and training expenditures for advanced AR systems are high. These technologies have the potential to exacerbate existing inequities in eye care, especially between high-resource and low-resource settings, if intentional measures are not taken to ensure equitable distribution. Determining the true benefit of AR in everyday practice will require cost-effectiveness studies and scalable implementation approaches.

Medico-legal and ethical considerations are equally significant. Concerns about accountability, data integrity, and physician autonomy arise as AR technologies increasingly impact clinical decisions. The ophthalmologist must remain the ultimate decision-maker, using AR as a supplement to clinical judgment rather than as a replacement. To keep patients’ and clinicians’ trust, algorithm design, data sources, and system restrictions must be transparent.

With the convergence of sophisticated imaging, telemedicine, and artificial intelligence, augmented reality may have an even greater impact in the future. Real-time AI-driven annotations, predictive analytics, and adaptive guidance systems have the potential to transform AR from a passive display tool into an active therapeutic companion. In ophthalmology, where conditions often require early detection and precise intervention, such cooperation could significantly improve outcomes.

In conclusion, augmented reality is a fascinating new area in ophthalmology since it provides fresh perspectives, insights, and opportunities for intervention. The potential benefits—for patients, surgeons, and trainees—are substantial, but zeal must be controlled by thorough evaluation and skillful implementation. As augmented reality continues to develop, ophthalmology may embrace it and help shape best practices for its integration into modern medicine. The problem that lies ahead is ensuring that this technology improves rather than diminishes the primary goal of ophthalmic care: preserving and restoring eyesight.

**Senior Lecturer Consuela-Mădălina Gheorghe, PhD, Philologist, Certified translator**

